# Supplementary material for: Downregulation of praja2 restrains endocytosis and boosts tyrosine kinase receptors in kidney cancer
Source: Commun Biol. 2024 Feb 20;7:208. doi: 10.1038/s42003-024-05823-4 (PMC10879500; doi:10.1038/s42003-024-05823-4)
Supplement: Supplementary file 2 — Supplementary Information [file 42003_2024_5823_MOESM2_ESM.pdf]

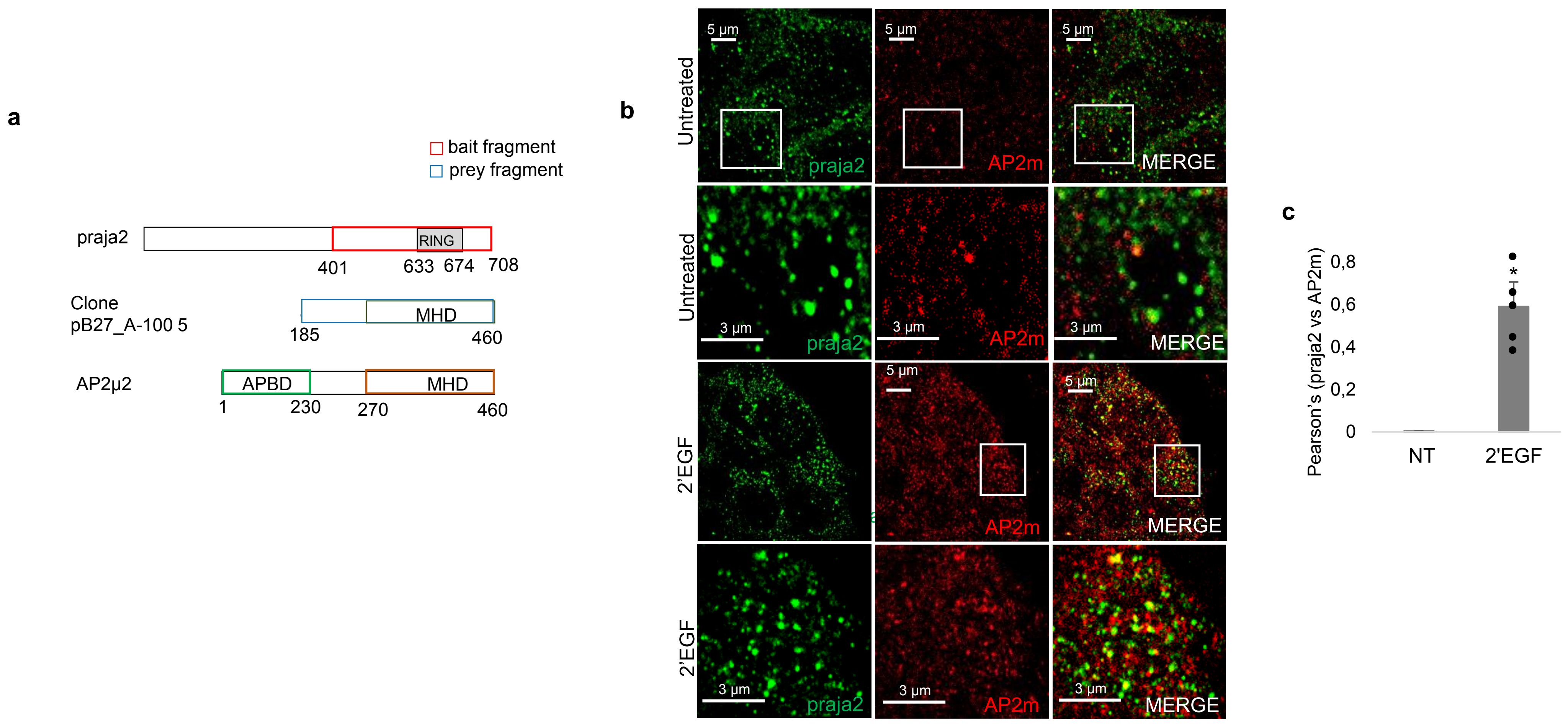

S1. (a) Schematic representation of praja2 domains. Highlighted in red is the bait fragment used for the yeast 2-hybrid screening. The prey fragment spanning residues 185-460 of AP-2 mu subunit is highlighted in blu.
(b) HeLa cells, left untreated or treated with EGF, were PFA-fixed and immunostained for praja2 and AP2m. Representative confocal images are shown. Scale bar (5 µM). **(c)** Pearson's coefficient and quantitative analysis of the experiments shown in c. A mean value of three independent experiments ± SD is shown. *P=0,014

Supplementary Figure 1

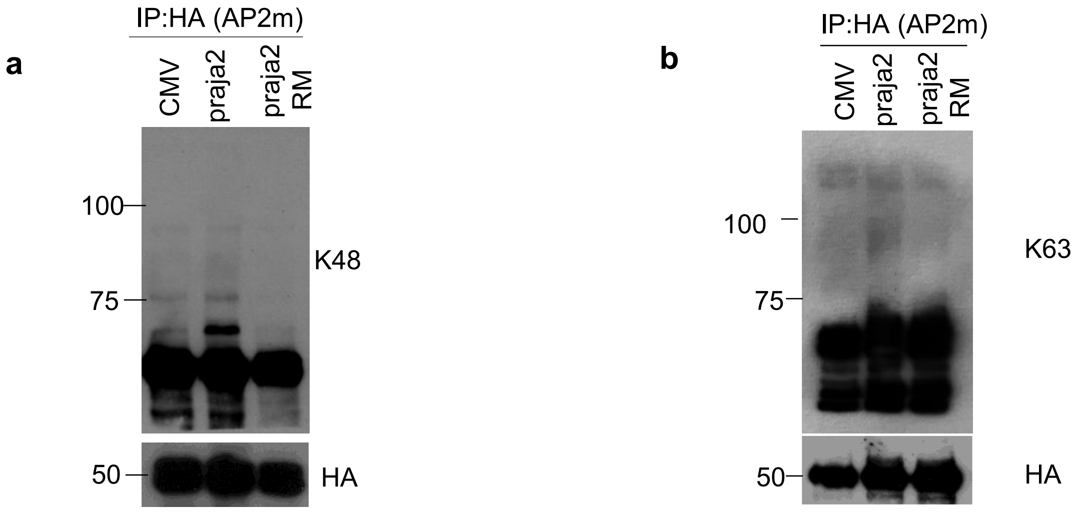

S2. (a) Immunoprecipitation of AP2m-HA from HEK293 cell lysates expressing AP2m-HA, Myc-ubiquitin and FLAG-praja2 or FLAG-praja2RM (inactive mutant). Precipitates were immunoblotted with anti-K8 (ubiquitinated AP2m) and anti-HA antibodies.
(b) Immunoprecipitation of AP2m-HA from HEK293 cell lysates expressing AP2m-HA, Myc-ubiquitin and FLAG-praja2 or FLAG-praja2RM (inactive mutant). Precipitates were immunoblotted with anti-K63 (ubiquitinated AP2m) and anti-HA antibodies.

Supplementary Figure 2

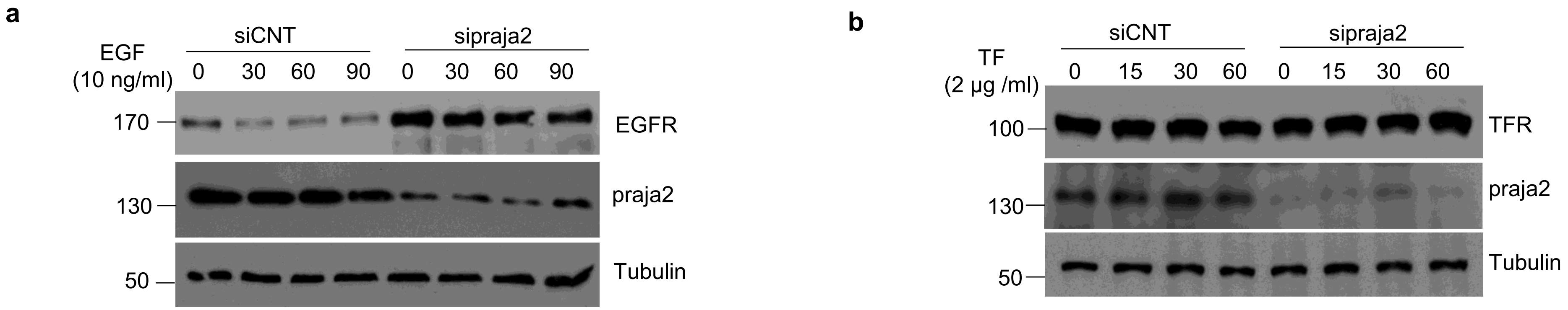

S3. a. HK2 cells were transfected with siRNAs (control or targeting praja2), serum-deprived overnight and treated with EGF (10 ng/ml) for indicated times. Lysates were immunoblotted for EGFR and praja2.

b. HeLa cells were transfected with siRNAs (control or targeting praja2), serum-deprived overnight and treated with transferrin (Tf, 2 µg/ml) up to 60 minutes. Lysates were immunoblotted for TfR and praja2.

Supplementary Figure 3

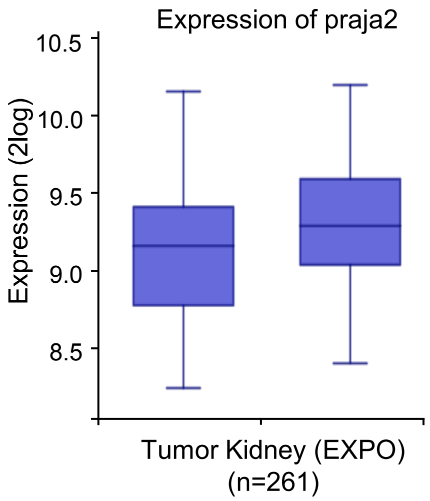

S4. Box plot showing relative expression (2log) of praja2 in normal (GSE18674) and tumors kidney tissues (GSE2109). Data were analyzed using “R2: Genomics Analysis and Visualization Platform” and one-way ANOVA test was performed ($p > 0.01$) for statistical analysis.

Supplementary Figure 4

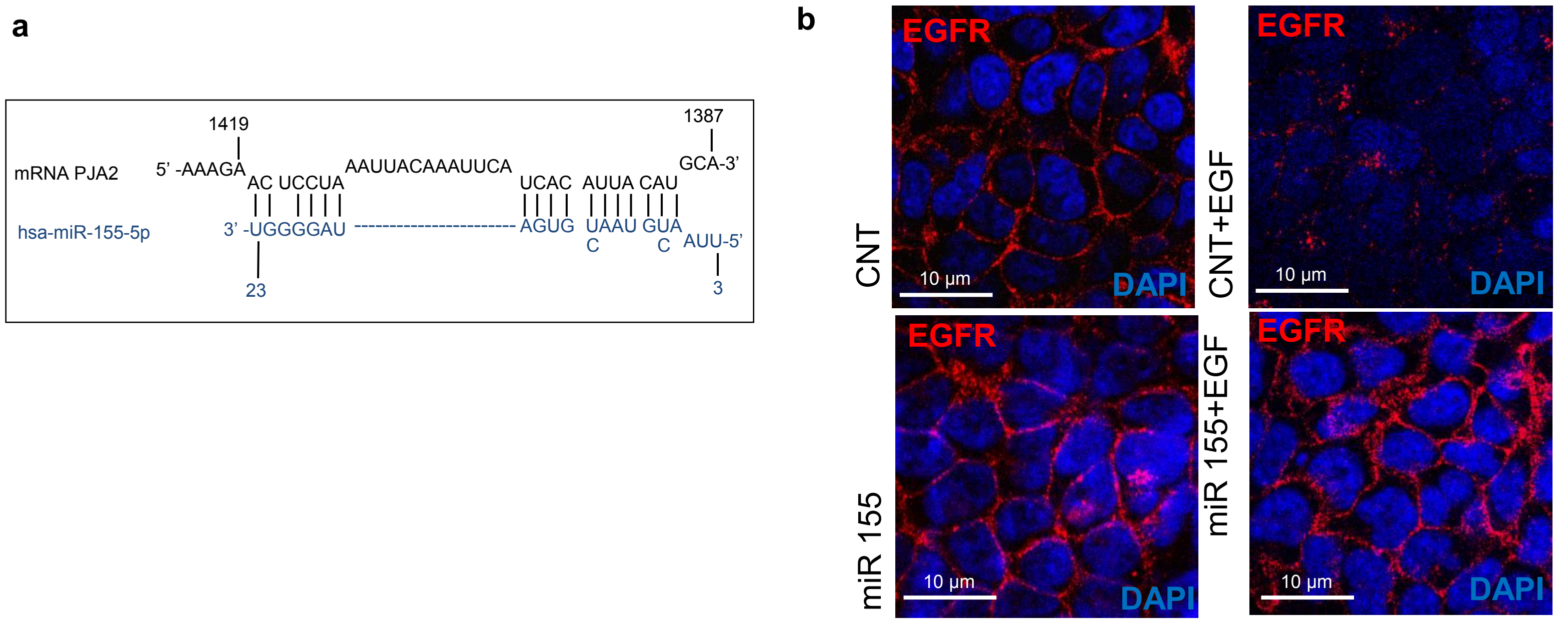

S5. (a) Hsa-miR-155-5p was predicted by target prediction programs intaRNA to align to positions 1387-1419 on the PJA2 3′UTR. Paired sequence alignment is marked by continuous lines. **(b)** HEK293 cells were transfected with miRNAs (control or 155), serum deprived and treated with EGF (10ng/µl) for 90 minutes. Cells were fixed and immunostained with anti EGFR antibody and DAPI. Representative confocal image are shown

Supplementary Figure 5

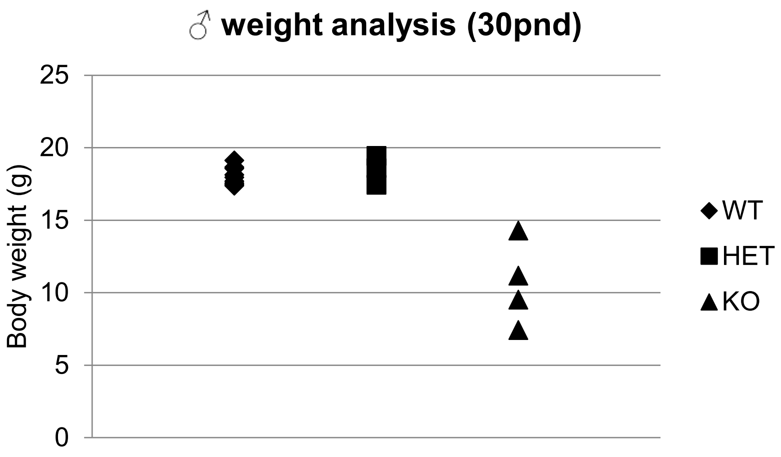

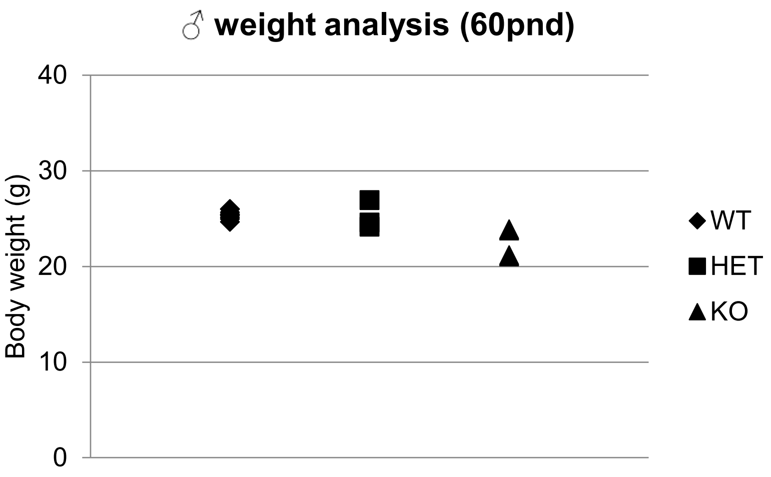

S6. Body weight of males Praja2 +/- , Praja2 -/- and control mice at 30 and 60 days after birth (n = 4 for each group)

Supplementary Figure 6

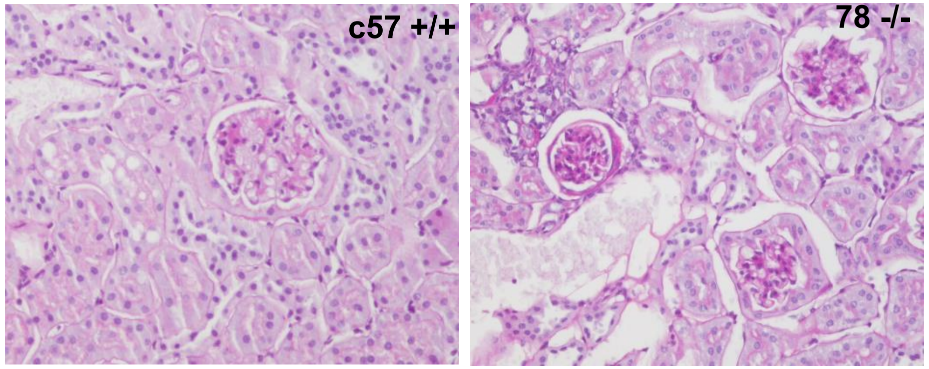

S7. Histological examination of kidney, mouse. Mild increase of PAS-positive mesangial matrix. Periodic acid – Schiff, 40x.

Supplementary Figure 7

Fig1b

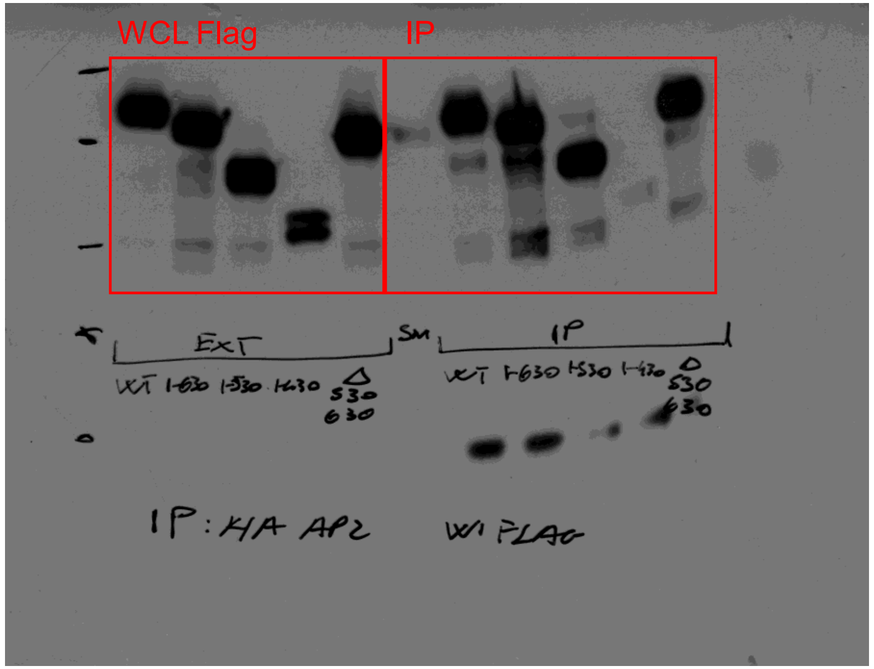

Fig1b

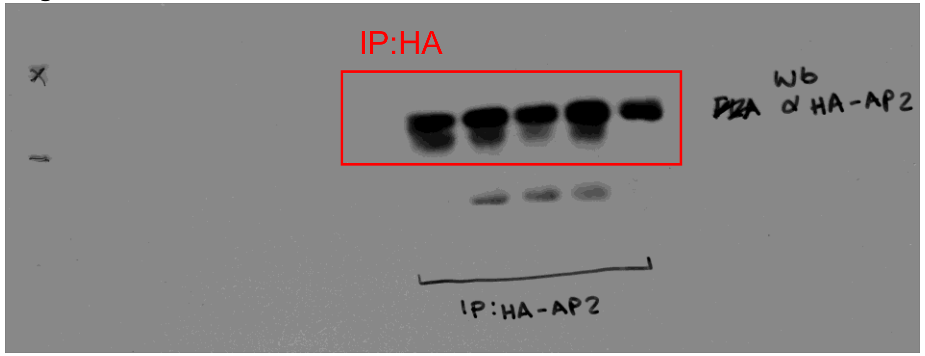

Fig1b

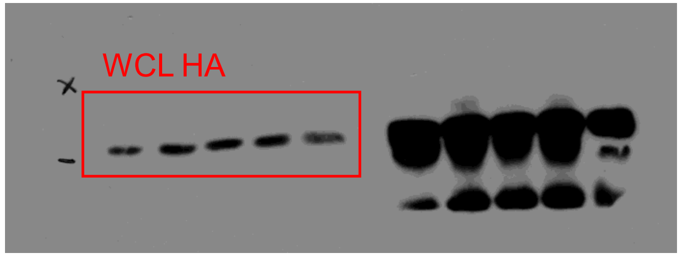

Fig1e

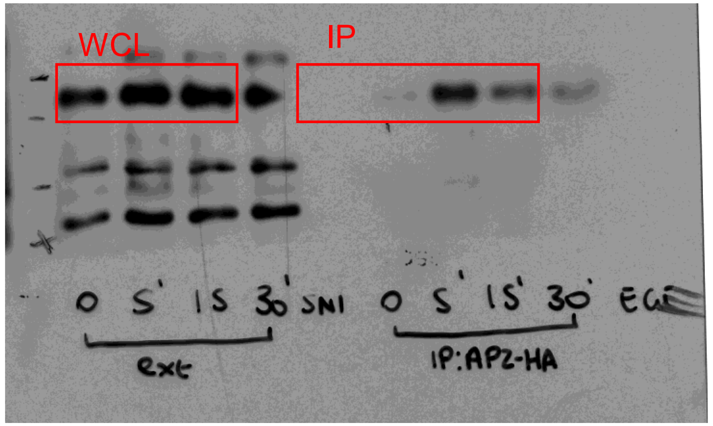

Fig1e

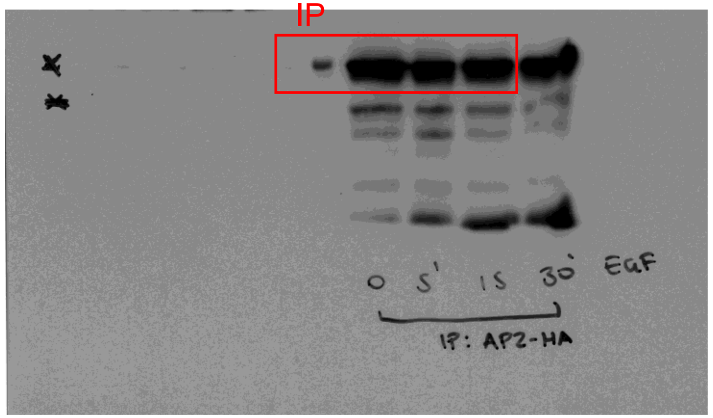

Fig1g

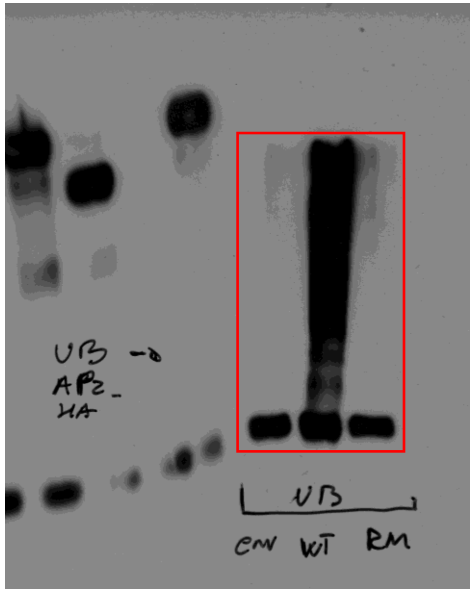

Fig1g

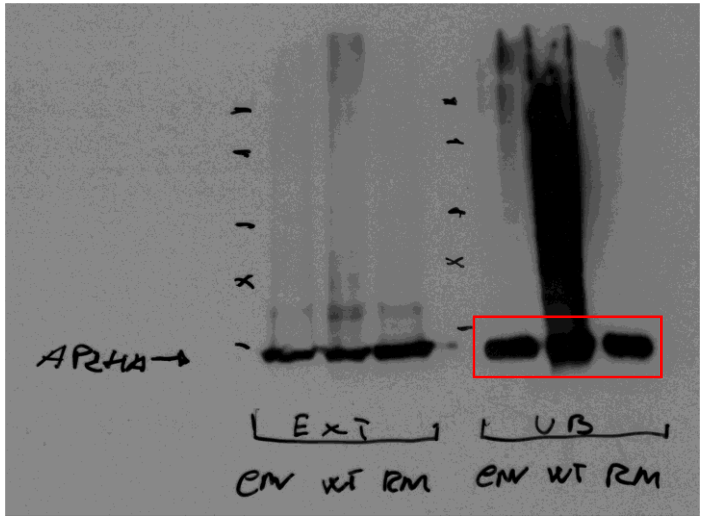

Fig1g

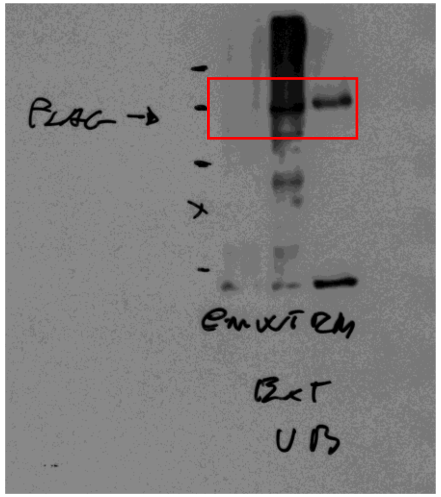

Fig1e

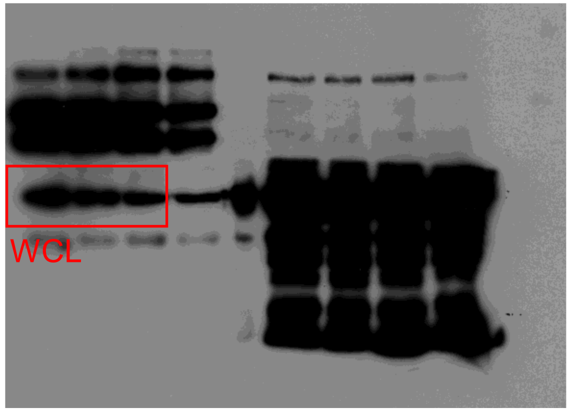

S8. Uncropped gels of the figures presented in Main and Supplementary Figures

Supplementary Figure 8

Fig1h

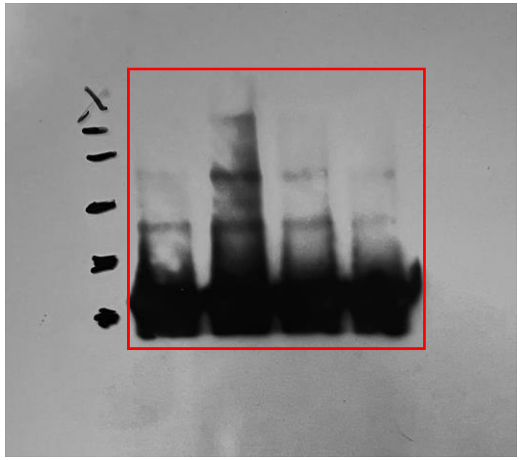

Fig1h

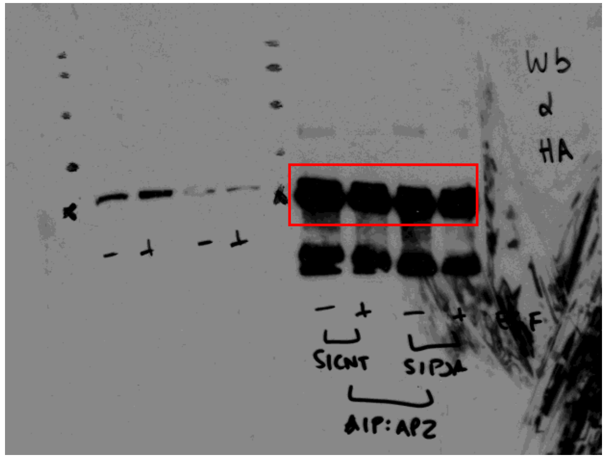

Fig2c

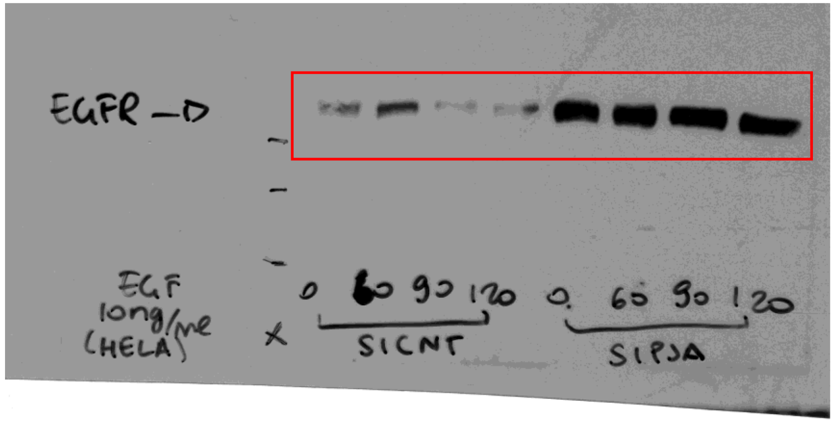

Fig2c

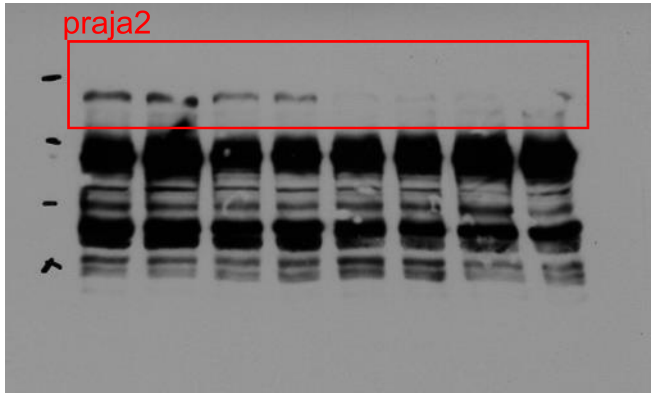

Fig2c

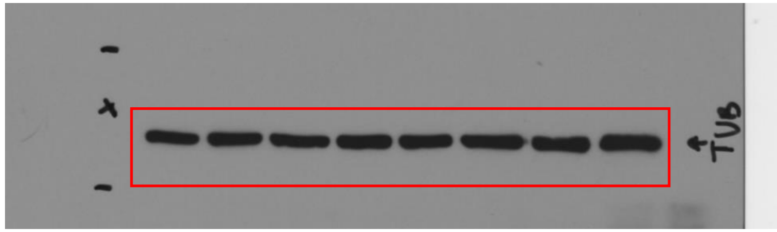

Fig2c

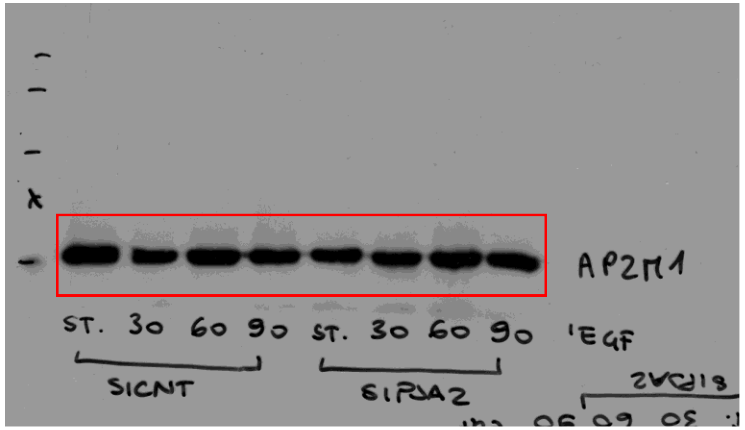

Fig3a

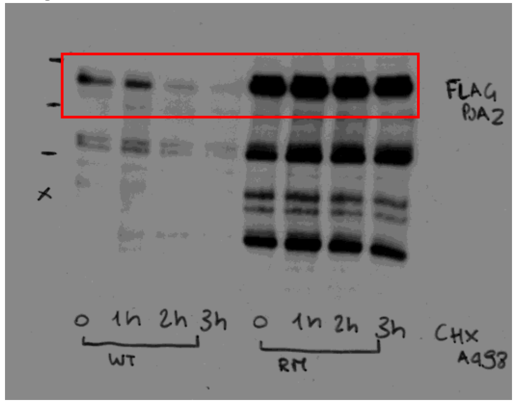

Fig3a

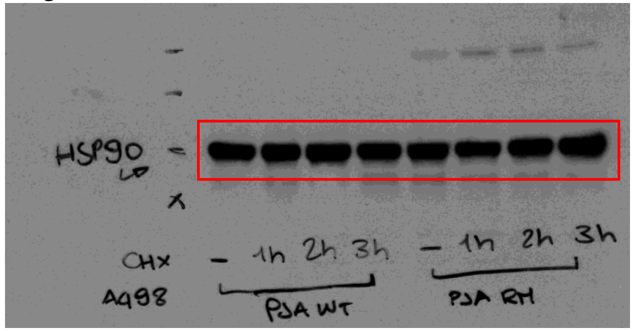

Fig3c

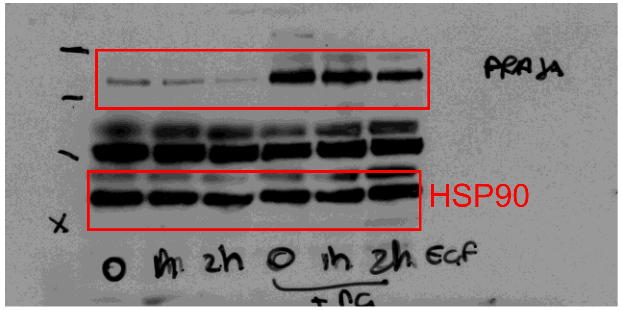

S8. Uncropped gels of the figures presented in Main and Supplementary Figures

Supplementary Figure 8

Fig3e

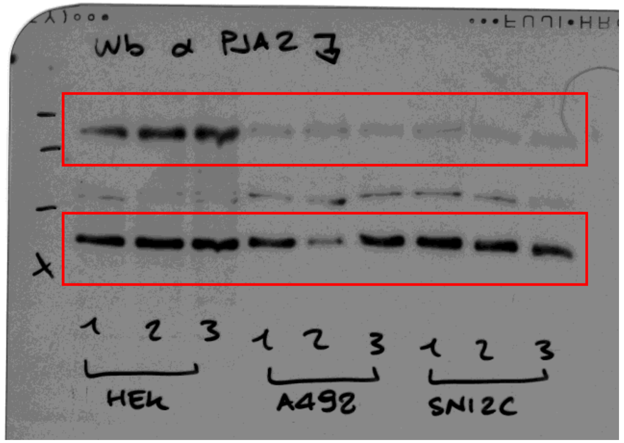

Fig3e

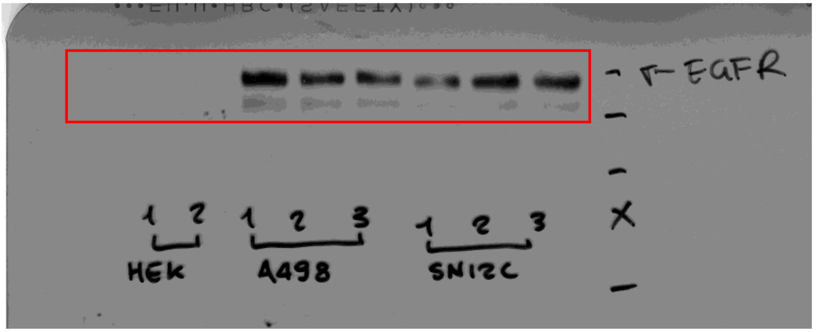

Fig3g

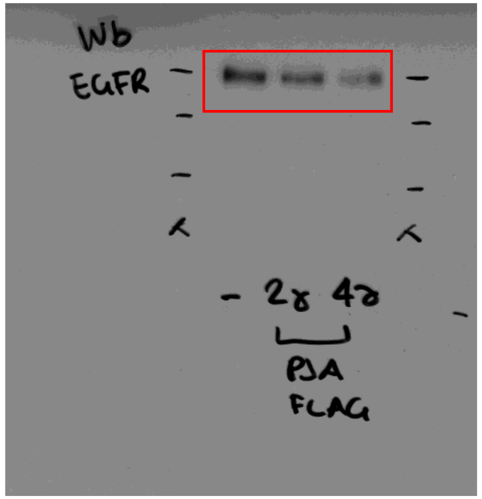

Fig3g

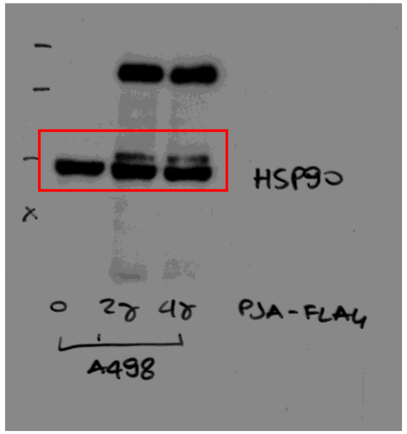

Fig3g

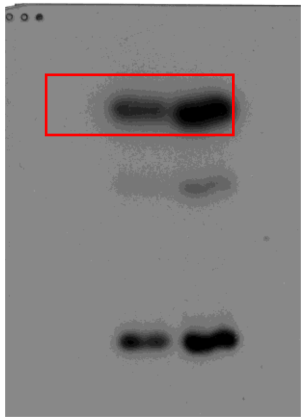

Fig3i

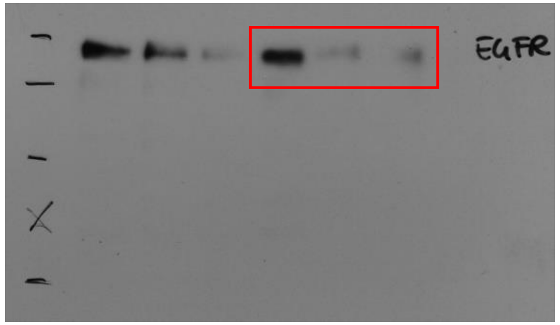

Fig3i

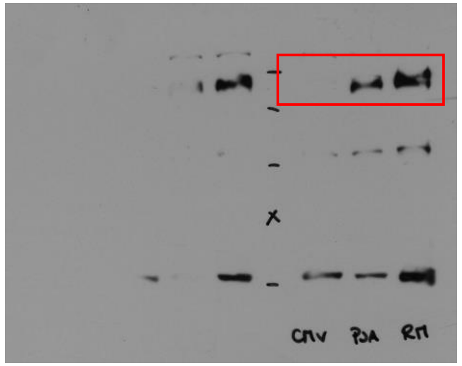

S8. Uncropped gels of the figures presented in Main and Supplementary Figures

Supplementary Figure 8

Fig4d

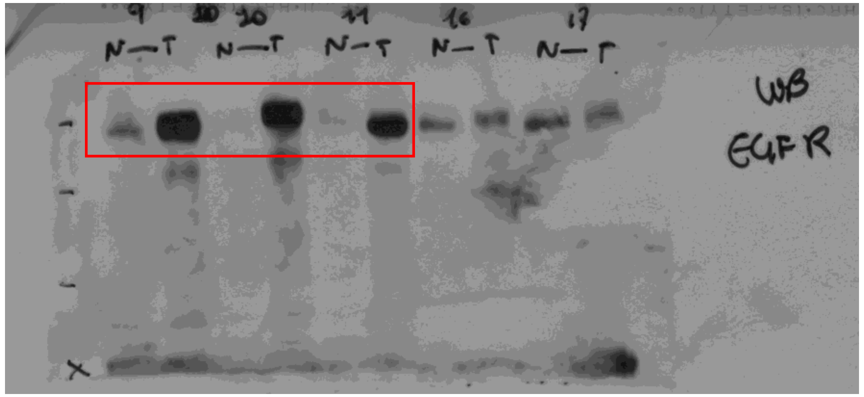

Fig4d

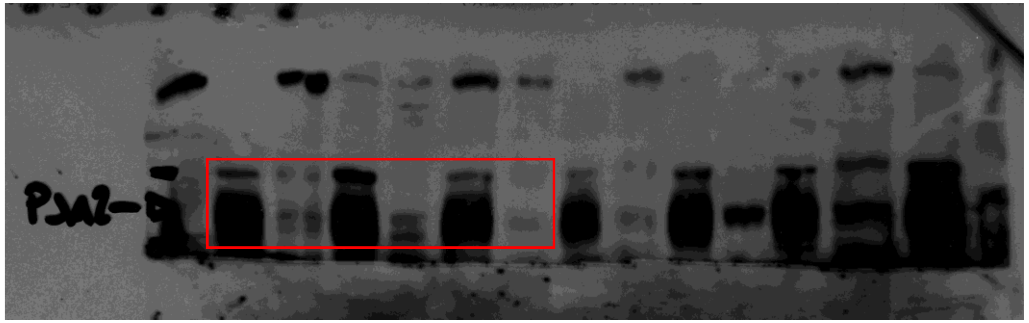

Fig4d

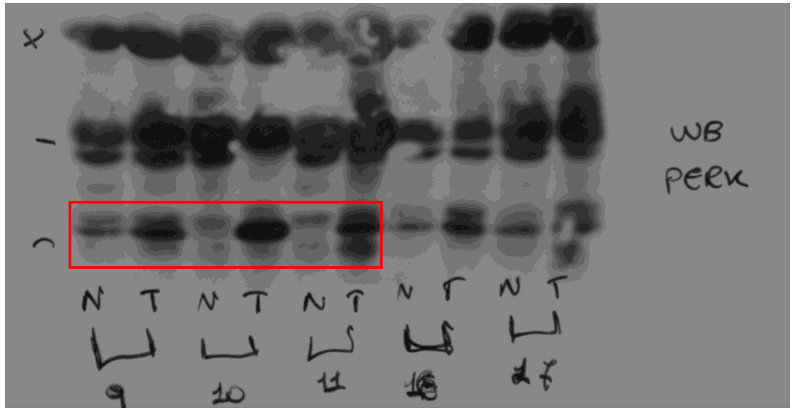

Fig4d

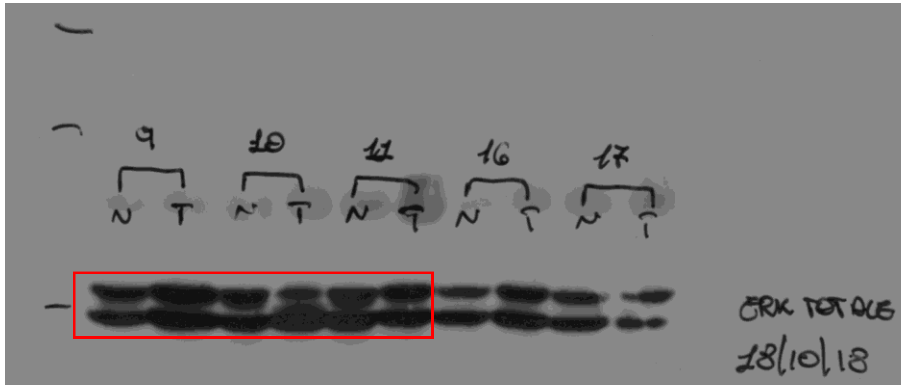

Fig6a

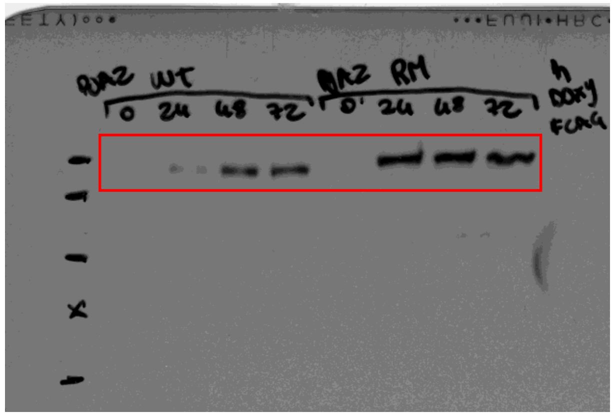

Fig6a

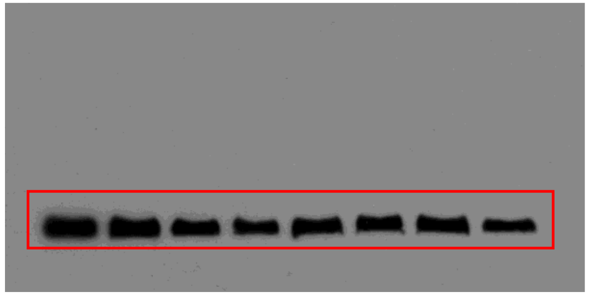

Fig5a

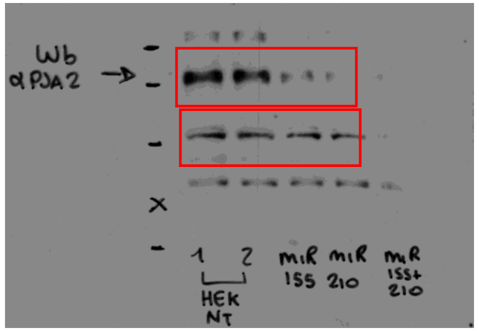

Fig6a

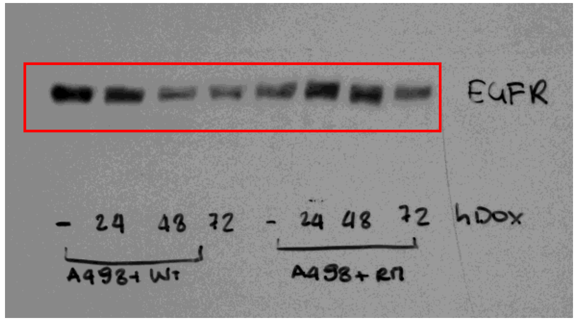

S8. Uncropped gels of the figures presented in Main and Supplementary Figures

Supplementary Figure 8

Fig S2a

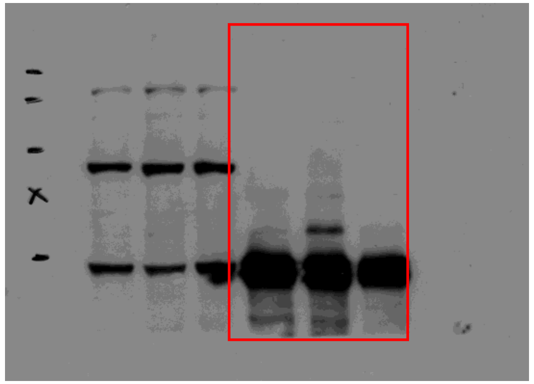

Fig S2a

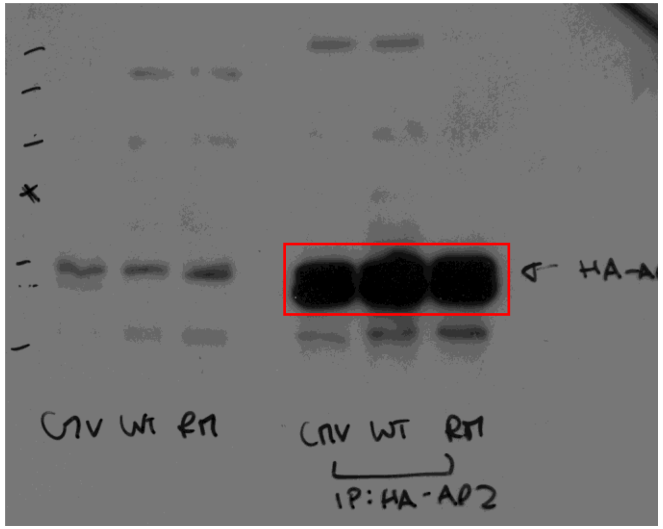

Fig S2b

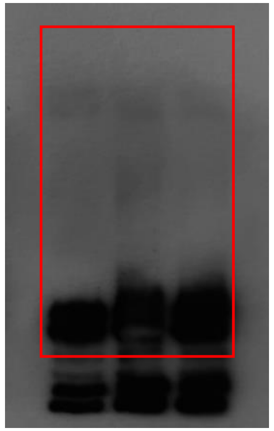

Fig S2b

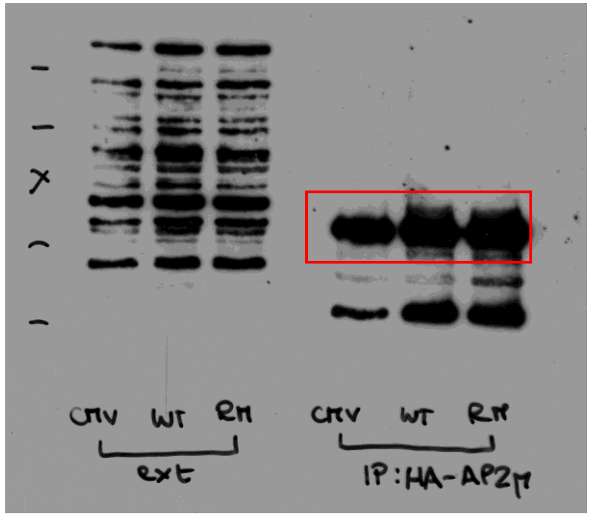

Fig S3a

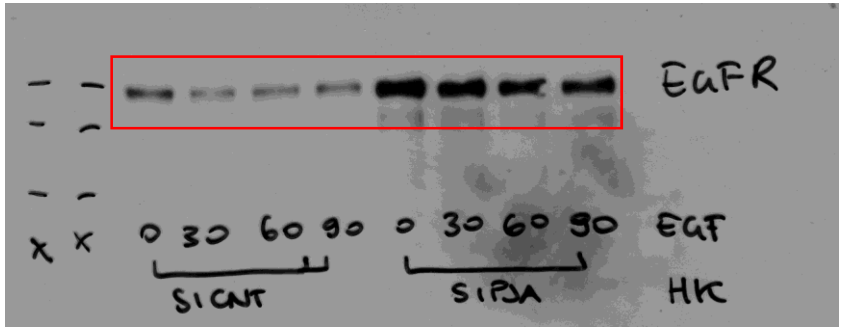

Fig S3a

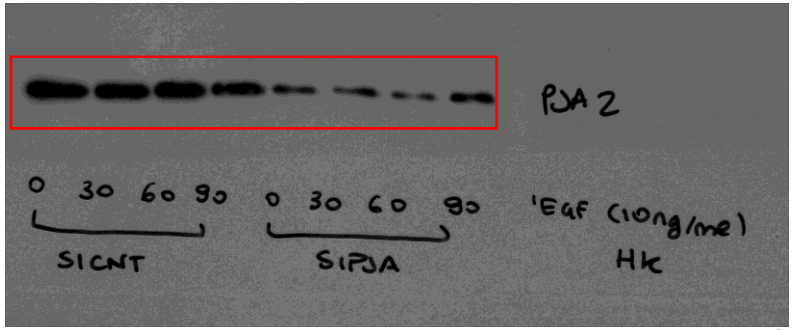

Fig S3a

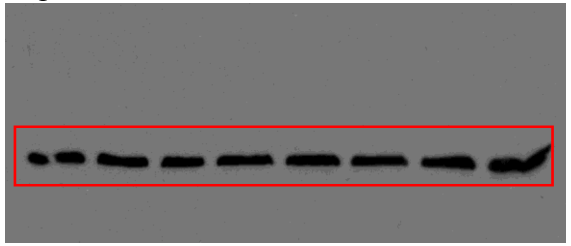

Fig S3b

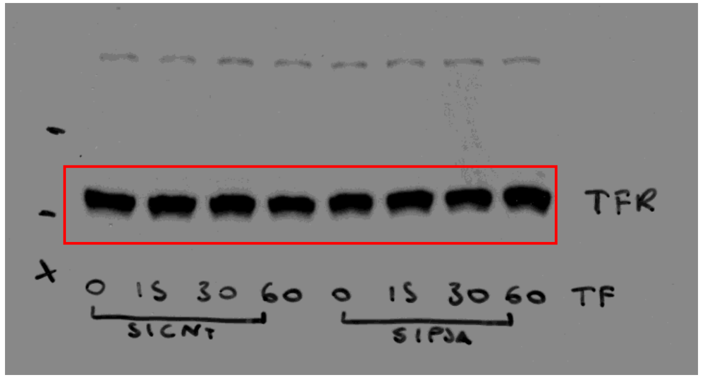

Fig S3b

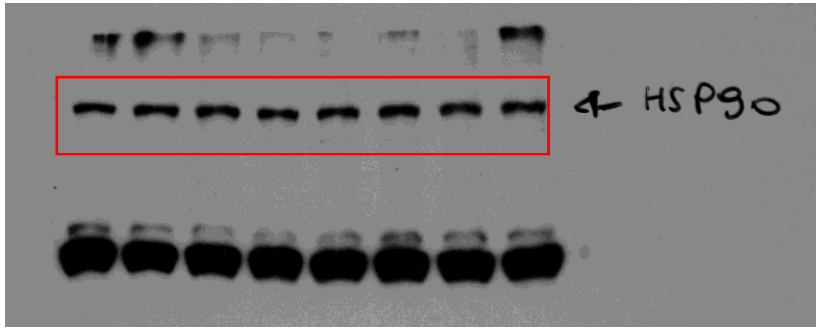

Fig S3b

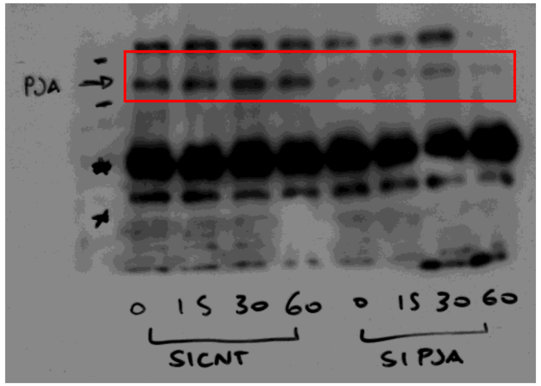

S8. Uncropped gels of the figures presented in Main and Supplementary Figures

Supplementary Figure 8

BD FACSDiva 8.0.1

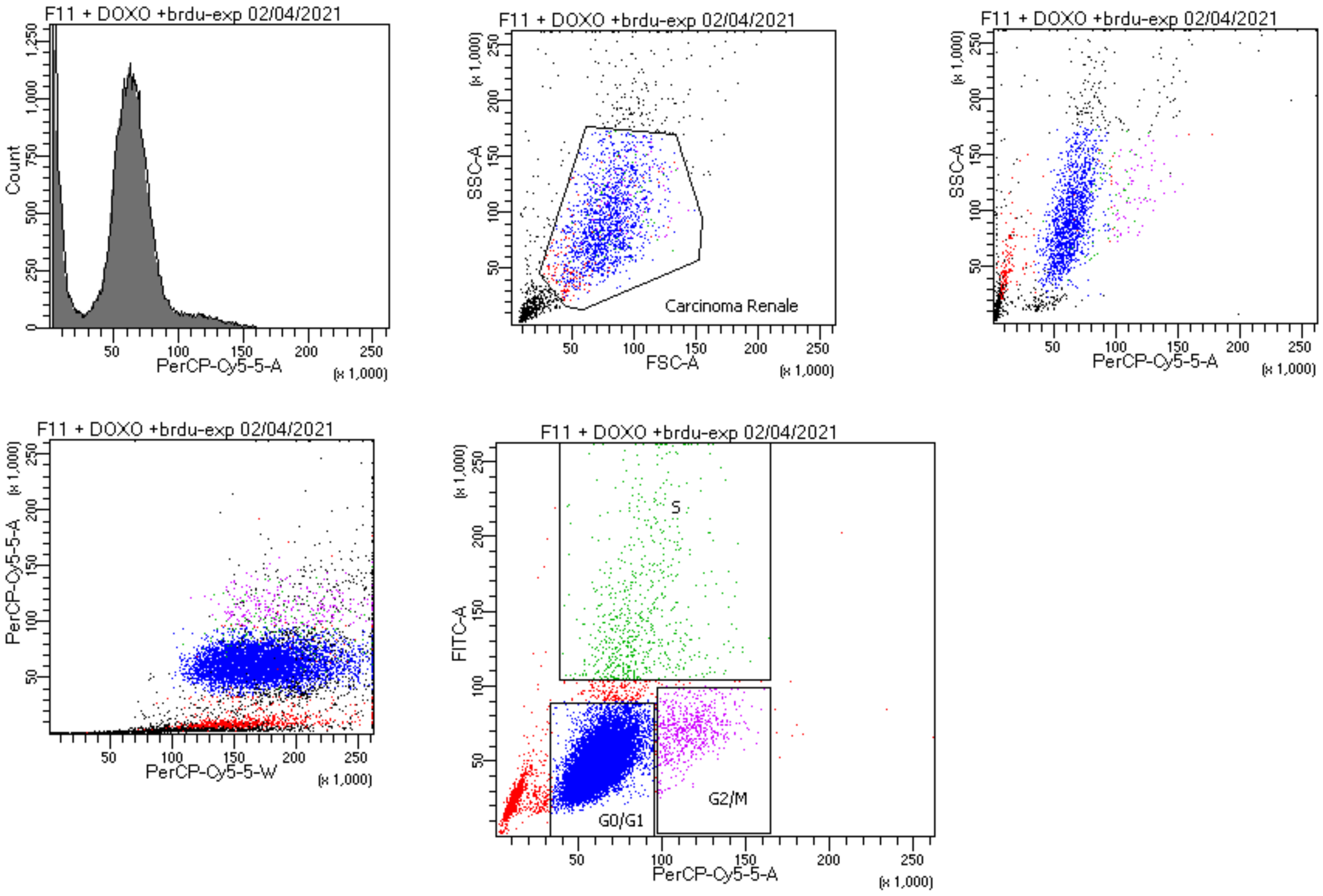

Supplementary Figure 9

S9. Gating strategy (FACS) data

Tube: exp 02/04/2021

Population	#Events	%Parent	%Total
All Events	49,010	####	100.0
Carcinoma Renale	34,454	70.3	70.3
S	1,302	3.8	2.7
G0/G1	28,693	83.3	58.5
G2/M	1,159	3.4	2.4

Experiment Name: Cloni in Carcinoma Renale ...
Specimen Name: F11 + DOXO +brdu
Tube Name: exp 02/04/2021
Record Date: Apr 2, 2021 3:59:52 PM
$OP: Administrator
GUID: 80a139ae-978c-4433-8bc4...

Population	#Events	%Parent	FITC-A Mean	PerCP-... Mean
Carcinoma Renale	34,454	70.3	52,153	60,177
S	1,302	3.8	153,945	86,960
G0/G1	28,693	83.3	49,040	61,302
G2/M	1,159	3.4	69,652	115,011

S9. Gating strategy (FACS) data

Supplementary Figure 9

BD FACSDiva 8.0.1

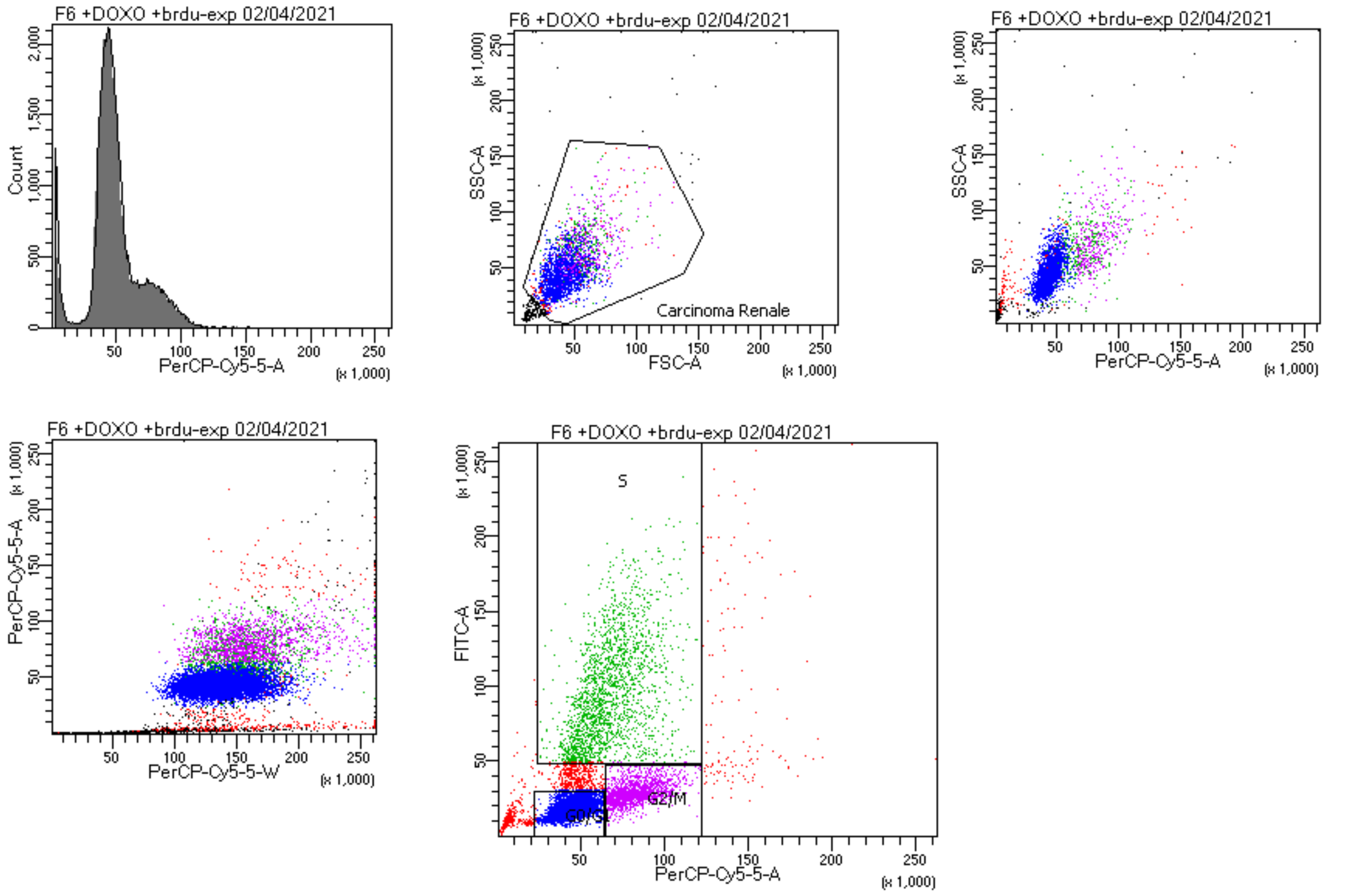

S9. Gating strategy (FACS) data

Supplementary Figure 9

BD FACSDiva 8.0.1

Tube: exp 02/04/2021

Population	#Events	%Parent	%Total
All Events	55,851	####	100.0
Carcinoma Renale	51,617	92.4	92.4
S	7,758	15.0	13.9
G0/G1	33,966	65.8	60.8
G2/M	5,904	11.4	10.6

Experiment Name: Cloni in Carcinoma Renale ...
Specimen Name: F6 +DOXO +brdu
Tube Name: exp 02/04/2021
Record Date: Apr 2, 2021 4:12:42 PM
$OP: Administrator
GUID: 0faef819-00f4-4b2b-9187-f...

Population	#Events	%Parent	FITC-A Mean	PerCP-... Mean
Carcinoma Renale	51,617	92.4	31,468	50,455
S	7,758	15.0	96,418	66,368
G0/G1	33,966	65.8	16,708	42,158
G2/M	5,904	11.4	28,355	81,925

S9. Gating strategy (FACS) data

Supplementary Figure 9
