# Supplementary material for: Downregulation of praja2 restrains endocytosis and boosts tyrosine kinase receptors in kidney cancer
Source: Commun Biol. 2024 Feb 20;7:208. doi: 10.1038/s42003-024-05823-4 (PMC10879500; doi:10.1038/s42003-024-05823-4)
Supplement: Supplementary file 3 — Description of Additional Supplementary Files [file 42003_2024_5823_MOESM3_ESM.pdf]

Description of Additional Supplementary Files

File name: Supplementary Data 1

Description: Name of gene products interacting with praja2

File name: Supplementary Data 2

Description: Clinical and histopathological data of patients enrolled in this study.

File name: Supplementary Data 3

Description: All data set obtained in this study
