# Supplementary material for: Downregulation of praja2 restrains endocytosis and boosts tyrosine kinase receptors in kidney cancer
Source: Commun Biol. 2024 Feb 20;7:208. doi: 10.1038/s42003-024-05823-4 (PMC10879500; doi:10.1038/s42003-024-05823-4)
Supplement: Supplementary file 7 — Reporting Summary [file 42003_2024_5823_MOESM7_ESM.pdf]

nature portfolio

Corresponding author(s): Antonio Feliciello

Last updated by author(s): November 23th, 2023

Reporting Summary

Nature Portfolio wishes to improve the reproducibility of the work that we publish. This form provides structure for consistency and transparency in reporting. For further information on Nature Portfolio policies, see our Editorial Policies and the Editorial Policy Checklist.

Statistics

For all statistical analyses, confirm that the following items are present in the figure legend, table legend, main text, or Methods section.

n/a	Confirmed	
☐	☒	The exact sample size (n) for each experimental group/condition, given as a discrete number and unit of measurement
☐	☒	A statement on whether measurements were taken from distinct samples or whether the same sample was measured repeatedly
☐	☒	The statistical test(s) used AND whether they are one- or two-sided<br>*Only common tests should be described solely by name; describe more complex techniques in the Methods section.*
☐	☒	A description of all covariates tested
☐	☒	A description of any assumptions or corrections, such as tests of normality and adjustment for multiple comparisons
☐	☒	A full description of the statistical parameters including central tendency (e.g. means) or other basic estimates (e.g. regression coefficient) AND variation (e.g. standard deviation) or associated estimates of uncertainty (e.g. confidence intervals)
☒	☐	For null hypothesis testing, the test statistic (e.g. F, t, r) with confidence intervals, effect sizes, degrees of freedom and P value noted<br>*Give P values as exact values whenever suitable.*
☒	☐	For Bayesian analysis, information on the choice of priors and Markov chain Monte Carlo settings
☒	☐	For hierarchical and complex designs, identification of the appropriate level for tests and full reporting of outcomes
☐	☒	Estimates of effect sizes (e.g. Cohen's d, Pearson's r), indicating how they were calculated

Our web collection on statistics for biologists contains articles on many of the points above.

Software and code

Policy information about availability of computer code

Data collection	Filtered reads were aligned using STAR v2.7.9a with standard parameters. Filtered reads were aligned on the human genome (assembly hg38) considering genes present in GenCode Release 37 (GRCh38.p13), while for KO Paja2 vs WT dataset, the sequences were aligned on mouse genome (release mm39), considering genes available in GenCode Release M32 (GRCm39). Quantification of expressed genes was performed using featureCounts(Liao et al, 2014) and differentially expressed genes were identified using DESeq2(Love et al, 2014). Functional analysis on differentially expressed genes was performed using Ingenuity Pathway Analysis (IPA, Qiagen). TCGA normal samples (n=72) and Kidney renal clear carcinoma (KIRC, n=533) primary tumors were analyzed with the UALCAN Platform.
Data analysis	Proteome Discoverer v. 2.4 software (Thermo Scientific), Mascot algorithm v. 2.6.1 (Matrix Science, UK), inBio Discover web tool (inBio Map Data Version 2021_04_07) , set of normalized gene expression data from kidney tumors (n=261, GSE2109) was analyzed and compared to normal tissues (n= 24, GSE18674) with "R2: Genomics Analysis and Visualization Platform".

For manuscripts utilizing custom algorithms or software that are central to the research but not yet described in published literature, software must be made available to editors and reviewers. We strongly encourage code deposition in a community repository (e.g. GitHub). See the Nature Portfolio guidelines for submitting code & software for further information.

Data

Policy information about availability of data

All manuscripts must include a data availability statement. This statement should provide the following information, where applicable:

- Accession codes, unique identifiers, or web links for publicly available datasets
- A description of any restrictions on data availability
- For clinical datasets or third party data, please ensure that the statement adheres to our policy

The RNA-seq raw data are publicly available in ArrayExpress repository under accession number: E-MTAB-11900(Doxy and NT datasets) and E-MTAB-13105 (KO Praja2 and WT datasets). The mass spectrometry proteomics data have been deposited to the ProteomeXchange Consortium via the PRIDE partner repository with the dataset identifier PXD034966.

Human research participants

Policy information about studies involving human research participants and Sex and Gender in Research.

Reporting on sex and gender	sex or gender are not considered ias a relevant parameter n the present study.
Population characteristics	Patients with diagnosis of clear cell renal cell carcinoma (ccRCC). Patients are 32 men and 21 women age from 43 to 78.
Recruitment	All tumors were retrieved from the files of the Department of Pathology, University of Naples "Federico II".
Ethics oversight	All patients gave their informed consent

Note that full information on the approval of the study protocol must also be provided in the manuscript.

Field-specific reporting

Please select the one below that is the best fit for your research. If you are not sure, read the appropriate sections before making your selection.

☒ Life sciences ☐ Behavioural & social sciences ☐ Ecological, evolutionary & environmental sciences

For a reference copy of the document with all sections, see nature.com/documents/nr-reporting-summary-flat.pdf

Life sciences study design

All studies must disclose on these points even when the disclosure is negative.

Sample size	We made no sample-size calculations. For each set of experiment sample size was extimated to be adequate based on the magnitude abd consistency of misurable differences among the groups analysed.
Data exclusions	On principle, data were only excluded for failed experiments.
Replication	Replicate experiments were successful
Randomization	Zebrafish were randomly assigned to the experimental groups
Blinding	Investigators were not blinded during experiments in vivo and in vitro

Reporting for specific materials, systems and methods

We require information from authors about some types of materials, experimental systems and methods used in many studies. Here, indicate whether each material, system or method listed is relevant to your study. If you are not sure if a list item applies to your research, read the appropriate section before selecting a response.

Materials & experimental systems

n/a	Involved in the study
☐	☒ Antibodies
☐	☒ Eukaryotic cell lines
☒	☐ Palaeontology and archaeology
☐	☒ Animals and other organisms
☒	☐ Clinical data
☒	☐ Dual use research of concern

Methods

n/a	Involved in the study
☒	☐ ChIP-seq
☐	☒ Flow cytometry
☒	☐ MRI-based neuroimaging

Antibodies

Antibodies used	Materials and Methods section provided with the manuscript contains information on all antibodies used in our study.
Validation	We used only commercially available, validate antibody.

Eukaryotic cell lines

Policy information about cell lines and Sex and Gender in Research

Cell line source(s)	Human embryonic kidney cell line (HEK293, ATCC), human cervical carcinoma cell line (HeLa, purchased from ATCC) and human kidney cell lines (A-498, HK-2, CaKi-1, purchased from ATCC)
Authentication	authentication of cell lines was carried out by ATTC
Mycoplasma contamination	Mycoplasm contamination was excluded using appropriate commercial kits (PCR and specific oligonucleotide primers) following manufacturer's procedures
Commonly misidentified lines (See ICLAC register)	No cell line used are listed in the database of commonly misidentified cell lines

Animals and other research organisms

Policy information about studies involving animals; ARRIVE guidelines recommended for reporting animal research, and Sex and Gender in Research

Laboratory animals	Tg(fli1:EGFP) zebrafish line, with green fluorescent vessels; C57BL/6J mice ,females and males.
Wild animals	No wild animals were used for experiments
Reporting on sex	The findings do not apply on sex
Field-collected samples	The study do not involve samples colleted from the field
Ethics oversight	All animal procedures were under the guidelines of Italian Ministry of Health and the study was approved by the Animal Care Authorities (Authorization number: 2014/0015838 and 924/2017-PR del 29/11/2017).

Note that full information on the approval of the study protocol must also be provided in the manuscript.

Flow Cytometry

Plots

Confirm that:

☒ The axis labels state the marker and fluorochrome used (e.g. CD4-FITC).

☒ The axis scales are clearly visible. Include numbers along axes only for bottom left plot of group (a 'group' is an analysis of identical markers).

☒ All plots are contour plots with outliers or pseudocolor plots.

☒ A numerical value for number of cells or percentage (with statistics) is provided.

Methodology

Sample preparation	A498 cells were processed according to the BrdU Flow Kit protocol BD Pharmingen, following the manufacturer's procedures
Instrument	BD FACS "CANTO II" FLOW CYTOMETER

Software	BD FACS DIVA software are used for the experiment
Cell population abundance	Cells were not sorted
Gating strategy	Forwars and side scatter gating; Pulse geometry gating; subsetting gating.

☐ Tick this box to confirm that a figure exemplifying the gating strategy is provided in the Supplementary Information.
